# Obituary: Vitalij Volodymirovitch SMYCHUK

**DOI:** 10.3389/jaws.2023.11449

**Published:** 2023-05-25

**Authors:** Andrew Charles de Beaux, Maciej Smietanski

**Affiliations:** ^1^ European Hernia Society, Edinburgh, United Kingdom; ^2^ European Hernia Society (EHS), Gdansk, Poland

**Keywords:** obituary, Ukraine, hernia surgeon, Russian invasion, hope

**FIGURE 1:**
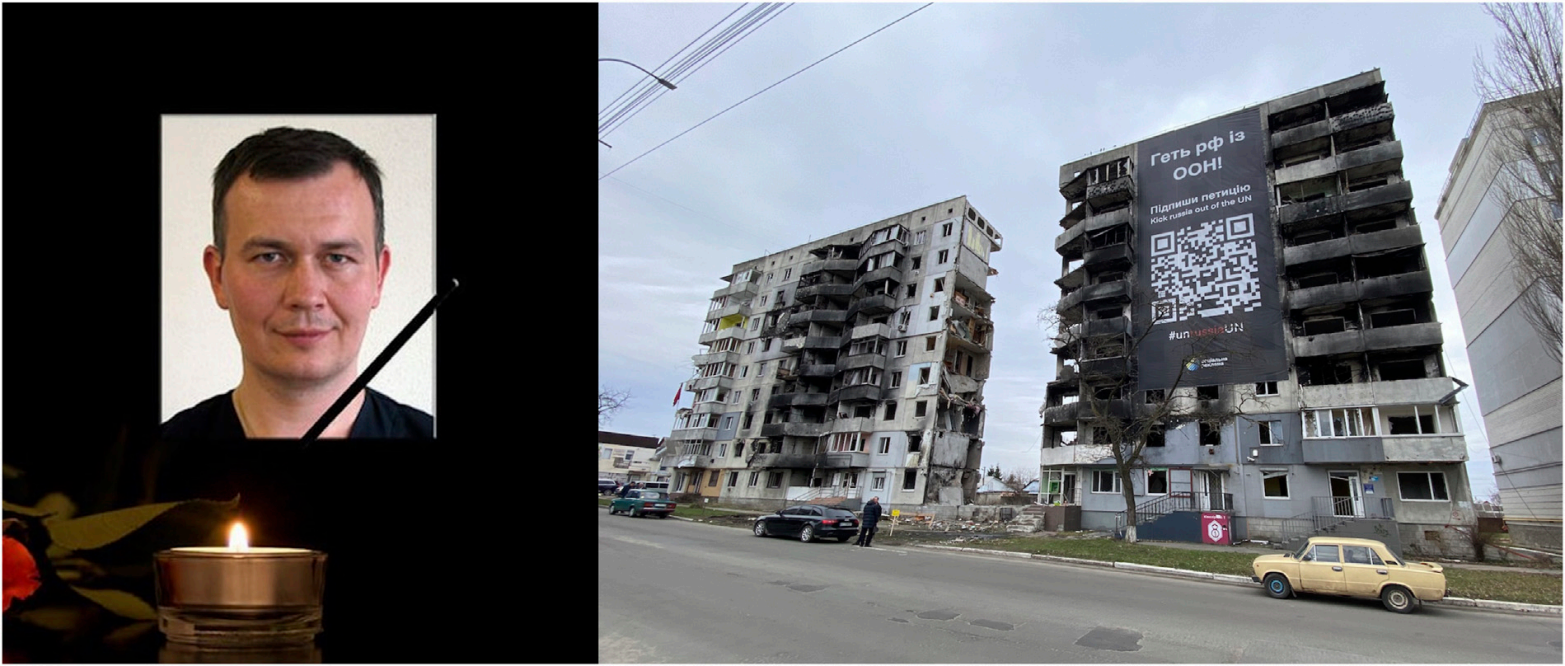
Vitalij Volodymirovitch SMYCHUK and the war damaged apartment block in which he was killed.

It is with great sadness that we tell of the circumstances of the death of our young Ukrainian colleague, Vitalij Volodymirovitch Smychuk (Figure 1). He was a true #herniafriend to those who met him, serving his family, his patients, the Ukrainian Hernia Society, and the European Hernia Society for which he had been a member for many years.

Vitalij was born and grew up in Eastern Europe. He graduated in medicine from Kyiv University. At the time of his death, he was a surgeon working at the Postgraduate University of Medicine in Kyiv, under Professor Yarislav Feleshtynsky. True to his name, Vitalij showed the vitality to so many aspects in his life. He was the author of 47 publications in the hernia field by the time of his death at the age of 39. His peers recognised his enthusiasm and passion, appointing his to the Ukrainian Hernia Society. And undoubtably destined to be a future president of that Society.

He lived with his wife and daughter, along with parents in Borodianka, a town to the north of Kyiv. Their home was in an apartment block, typical of the region. On the fateful night, the air raid sirens sounded yet again, announcing yet another Russian missile assault. He awoke his family and parents to get them to the shelter in the basement of their apartment block. This was beneath the apartments slightly to the side of where he lived. His parents said that they would be too slow and to go without them. So he ran with his wife and child to the basement shelter. A short while later, half a ton of incendiary explosives fell on the neighbouring apartment block, under which was the shelter, creating a huge explosive inferno. Tragically, the three of them died (along with a number of others who also sought shelter), while his parents survived in the neighbouring apartment, which was badly damaged (Figure 1). His wife and child were never identified in the aftermath of the attack, but his body, or what was left of it, was identified from DNA.

It was our privilege to visit the site of the attack almost a year later. Words cannot express all the emotions we had that day, and they will remain with us for years to come. We laid a small token at the door to his apartment, on behalf of everyone in the European Hernia Society.

With time, the ruined apartment block will be demolished and no doubt rebuilt. Life and happiness will return to the region. But it is our responsibility to record this atrocious event, and its associated senseless loss of life, forever. We are humbled to be able to do this. Saddened beyond measure at this Russian atrocity. We hope the world will not forget.

